# A high-throughput method for isolation of salicylic acid metabolic mutants

**DOI:** 10.1186/1746-4811-6-21

**Published:** 2010-09-23

**Authors:** George Marek, Ryan Carver, Yezhang Ding, Deepak Sathyanarayan, Xudong Zhang, Zhonglin Mou

**Affiliations:** 1 Department of Microbiology and Cell Science, University of Florida, P.O. Box 110700, Gainesville, FL, 32611, USA

## Abstract

**Background:**

Salicylic acid (SA) is a key defense signal molecule against biotrophic pathogens in plants. Quantification of SA levels in plants is critical for dissecting the SA-mediated immune response. Although HPLC and GC/MS are routinely used to determine SA concentrations, they are expensive and time-consuming. We recently described a rapid method for a bacterial biosensor *Acinetobacter *sp. ADPWH_*lux*-based SA quantification, which enables high-throughput analysis. In this study we describe an improved method for fast sample preparation, and present a high-throughput strategy for isolation of SA metabolic mutants.

**Results:**

On the basis of the previously described biosensor-based method, we simplified the tissue collection and the SA extraction procedure. Leaf discs were collected and boiled in Luria-Bertani (LB), and then the released SA was measured with the biosensor. The time-consuming steps of weighing samples, grinding tissues and centrifugation were avoided. The direct boiling protocol detected similar differences in SA levels among pathogen-infected wild-type, *npr1 *(nonexpressor of pathogenesis-related genes), and *sid2 *(SA induction-deficient) plants as did the previously described biosensor-based method and an HPLC-based approach, demonstrating the efficacy of the protocol presented here. We adapted this protocol to a high-throughput format and identified six *npr1 *suppressors that accumulated lower levels of SA than *npr1 *upon pathogen infection. Two of the suppressors were found to be allelic to the previously identified *eds5 *mutant. The other four are more susceptible than *npr1 *to the bacterial pathogen *Pseudomonas syringae *pv. *maculicola *ES4326 and their identity merits further investigation.

**Conclusions:**

The rapid SA extraction method by direct boiling of leaf discs further reduced the cost and time required for the biosensor *Acinetobacter *sp. ADPWH_*lux*-based SA estimation, and allowed the screening for *npr1 *suppressors that accumulated less SA than *npr1 *after pathogen infection in a high-throughput manner. The highly efficacious SA estimation protocol can be applied in genetic screen for SA metabolic mutants and characterization of enzymes involved in SA metabolism. The mutants isolated in this study may help identify new components in the SA-related signaling pathways.

## Background

Salicylic acid (SA) is a key signaling molecule in plant defense against biotrophic pathogens [1,2]. Upon pathogen attack, SA accumulates in plant cells [3,4]. Prevention of SA accumulation leads to disease susceptibility [5], whereas treatment with SA confers resistance to a variety of biotrophic pathogens [6,7]. Thus, understanding the mechanisms underlying SA accumulation is critical in the study of plant immunity.

Nawrath and Métraux [8] performed a genetic screen for Arabidopsis mutants that do not accumulate SA after pathogen infection and identified two genetic loci, *SID1*/*EDS5 *and *SID2*/*EDS16*, which were later shown to encode a chloroplast MATE (multidrug and toxin extrusion) transporter [9] and an SA biosynthetic enzyme ICS1 (isochorismate synthase) [10], respectively. In the screen, Nawrath and Métraux used an HPLC-based method to quantify the SA levels in the pathogen-infected leaf tissues from about 4,500 individual M_2 _plants. Because the HPLC-based method involves extraction of SA in organic solvents, evaporation of organic solvents, chromatographic purification and detection by fluorescence spectroscopy [11,12], it is extremely costly and time-consuming. To screen for more SA metabolic mutants, a much faster and less expensive method is needed.

Huang *et al*. recently developed an SA biosensor, named *Acinetobacter *sp. ADPWH_*lux *[13]. This strain is derived from *Acinetobacter *sp. ADP1, and contains a chromosomal integration of a salicylate-inducible *luxCDABE *operon. The operon encodes a luciferase (LuxA and LuxB) and the enzymes that produce its substrate (LuxC, LuxD and LuxE) so cells that express the cluster emit the 490-nm light spontaneously [14]. The biosensor is highly specific to SA, methyl-SA, and the synthetic SA derivative acetylsalicylic acid [13], thus suitable for the quantification of SA from crude plant extracts.

We previously described an approach for the simultaneous quantification of free and glucose conjugated SA from Arabidopsis leaf extracts using *Acinetobacter *sp. ADPWH_*lux *[15]. Here we present a further shortened protocol for the estimation of SA levels in pathogen-infected leaf tissue. Using the protocol described, we have performed a genetic screen for suppressors of the *npr1 *(nonexpressor of pathogenesis-related genes) mutant that hyperaccumulates SA during pathogen infection [16,17].

## Results

### Rapid Extraction of SA by Direct Boiling of Leaf Discs

**Figure 1:**
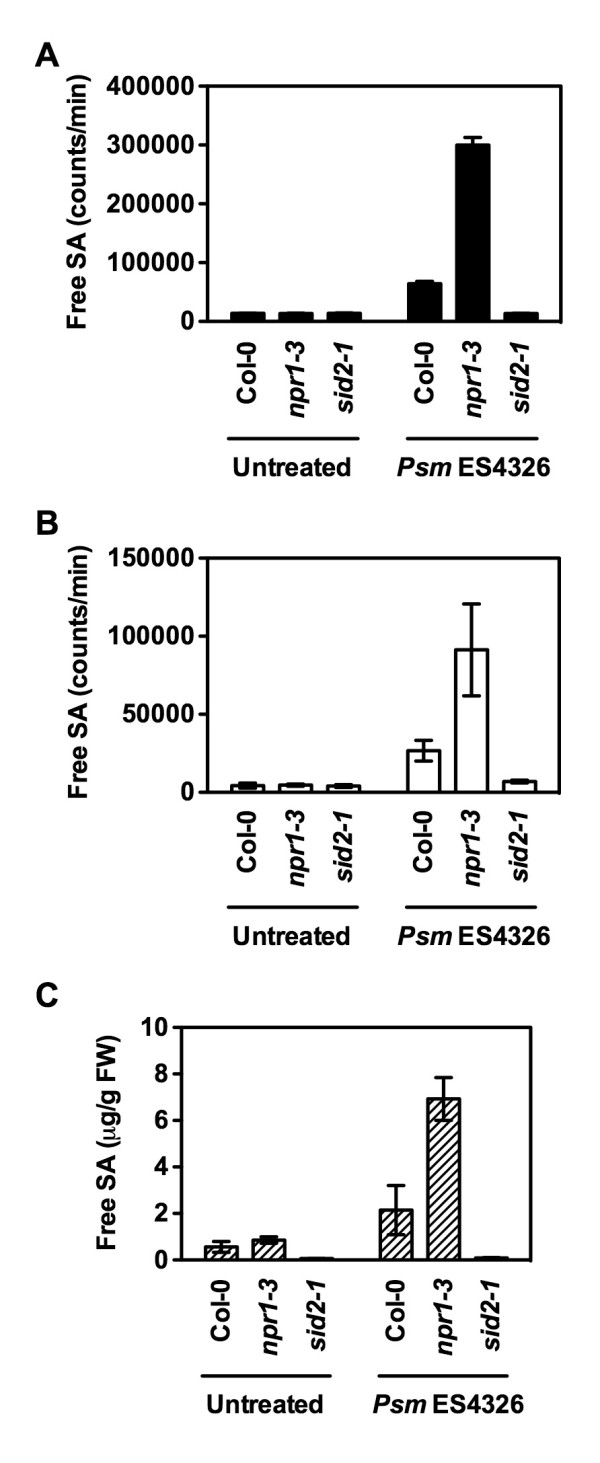
**The direct boiling method in comparison with the previously described biosensor- and HPLC-based methods**. (**A**) Luminescence from *Psm *ES4326-infected Col-0, *npr1 *and *sid2 *detected by the direct boiling method. (**B**) Luminescence from *Psm *ES4326-infected Col-0, *npr1 *and *sid2 *detected by the previously described biosensor-based method. (**C**) Free SA levels in *Psm *ES4326-infected Col-0, *npr1 *and *sid2 *detected by the HPLC-based method. Values are the mean of 8 samples (**A**), 6 samples read in triplicate (**B**), and 4 samples (**C**) with standard deviation.

The method we previously described comprises leaf tissue collection (weighing samples), grinding, extraction in LB or acetate buffer, and centrifugation [15]. The resulting crude leaf extract is then mixed with a culture of the SA biosensor in a 96-well cell culture plate, and incubated at 37°C for one hour. The luminescence is then determined. Compared with the conventional HPLC or GC/MS method [11,18], the biosensor-based method is much faster and requires little tissue (as few as 2-3 leaves) [15]. However, the tissue collection (especially weighing samples) and the extraction procedure are still time-consuming. To search for an even faster method, we collected leaf discs (0.7 cm in diameter, omitting weighing the samples) with a hole punch from the bacterial pathogen *Pseudomonas syringae *pv. *maculicola *(*Psm*) ES4326-infected leaves of wild-type Col-0, *npr1 *and *sid2 *plants. We used pathogen-infected Col-0, *npr1 *and *sid2 *plants as samples because they contain significantly different levels of SA [8,17]. Each leaf disc was placed in 200 μL of LB in a 1.5-mL eppendorf tube, and boiled at 95°C for 20 minutes. The LB extracts were cooled down to room temperature, and then mixed with the SA biosensor and incubated at 37°C for one hour. The luminescence was then determined. We found that SA was readily extracted by the direct boiling of leaf discs. As shown in Figure 1A, the pathogen-infected Col-0 and *npr1 *samples had significantly higher luminescence than uninfected samples, whereas the *sid2 *samples just had background luminescence regardless of whether they were infected or not. Furthermore, the luminescence emitted by the infected *npr1 *samples was significantly stronger than that emitted by the infected Col-0 samples (Figure 1A). The differences among Col-0, *npr1 *and *sid2 *were very similar to those revealed by the previously described biosensor-based method and the HPLC-based approach (Figures 1B and 1C). These results suggest that the direct boiling method is able to detect the differences in SA levels among different genotypes, thus suitable for genetic screens for mutants with altered SA levels.

### High-Throughput Screening for SA Metabolic Mutants

**Figure 2:**
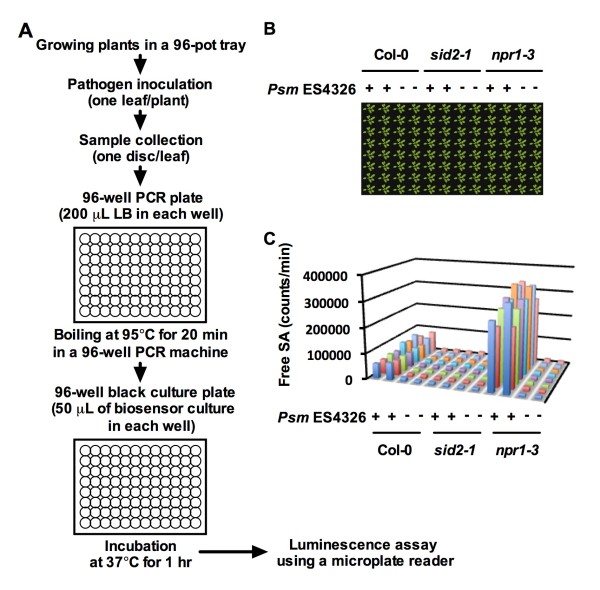
**High-throughput strategy for isolation of SA metabolic mutants**. (**A**) Schematic of the "96-pot tray/96-well PCR plate/96-well culture plate" screen strategy. (**B**) Col-0, *npr1 *and *sid2 *plants grown in a 96-cell tray treated with or without *Psm *ES4326. (**C**) Luminescence from the Col-0, *npr1 *and *sid2 *plants in (**B**) treated with or without *Psm *ES4326.

We adapted the direct boiling protocol to a high-throughput format competent for genetic screens. The strategy is schematically described in Figure 2A and a detailed protocol is presented in Additional file 1. Again, we used Col-0, *npr1 *and *sid2 *to test the efficacy of the strategy. Seedlings of Col-0, *npr1 *or *sid2 *were transplanted into one third (32 pots) of a 96-pot tray (Figure 2B). Three weeks later, half of the plants of each genotype were inoculated with *Psm *ES4326 (Figure 2B). To save the time spent on inoculation, only one leaf on each plant was inoculated. Twenty-four hours later, a leaf disc from each inoculated leaf was collected using a hole punch, and placed into 200 μL of LB in a corresponding well of a 96-well PCR plate. After all 96 leaf discs (from the 96 plants in Figure 2B) were collected and placed into the 96 wells, the PCR plate was put in a PCR machine and heated at 95°C for 20 minutes. The extracts were cooled down to room temperature, and 50 μL of each extract was added into a corresponding well in a black 96-well culture plate loaded with 50 μL of a freshly prepared biosensor culture in each well. After incubation at 37°C for 1 hour in the dark, luminescence was assayed using a microplate luminometer. As shown in Figure 2C, the differences in SA levels among the three genotypes were clearly detected using the "96-pot tray/96-well PCR plate/96-well culture plate" format.

We then attempted to set up a mutant screen aimed at identifying new components involved in regulating SA accumulation. Since the *npr1 *mutant accumulates significantly higher levels of SA than wild type during pathogen infection, and NPR1 is a key positive regulator of SA-mediated immune responses [19-21], we reasoned that *npr1 *suppressors, which accumulate less pathogen-induced SA than *npr1*, would help uncover important regulators of plant immunity. We therefore decided to use the *npr1 *mutant as starting material for the screen.

**Figure 3:**
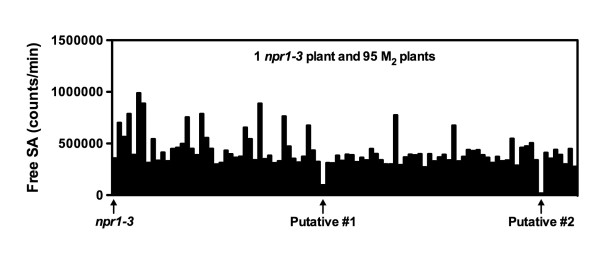
**Luminescence from a randomly picked tray of plants in the primary screen**. Luminescence from *Psm *ES4326-infected M_2 _plants was determined using the direct boiling method in a "96-pot tray/96-well PCR plate/96-well culture plate" format. Arrows indicate the putative *npr1 *suppressors.

One gram of *npr1 *seeds were treated with ethyl methanesulfonate (EMS) and sown on soil. M_2 _seeds were collected in pools when the M_1 _plants matured. After germination, M_2 _seedlings were transplanted into 96-pot trays and screened as described in Figure 2A. So far, approximately 10,000 M_2 _plants have been screened. Figure 3 shows the luminescence from the plants in a randomly selected tray in the primary screen. The primary screen has identified 35 putative *npr1 *suppressors.

**Figure 4:**
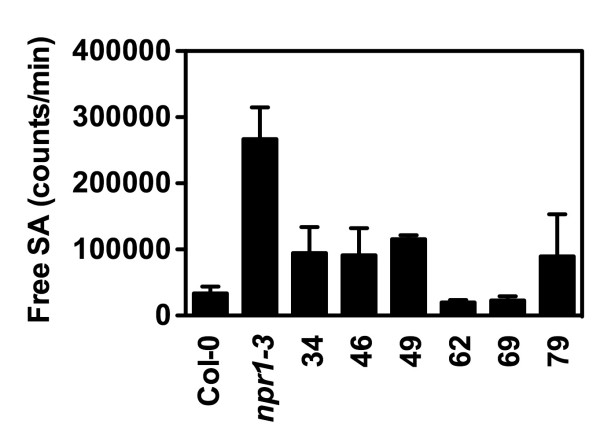
**Luminescence from six putative *npr1 *suppressors in the secondary screen**. Luminescence from *Psm *ES4326-infected M_3 _plants of six putative *npr1 *suppressors was determined using the direct boiling method.

The putative suppressors were re-screened using the direct boiling protocol. In the secondary screen, three M_3 _plants from each putative suppressor were assayed. As shown in Figure 4, six putative suppressors had lower luminescence than *npr1*.

### Confirmation of the Putative SA Metabolic Mutants Using HPLC

**Figure 5:**
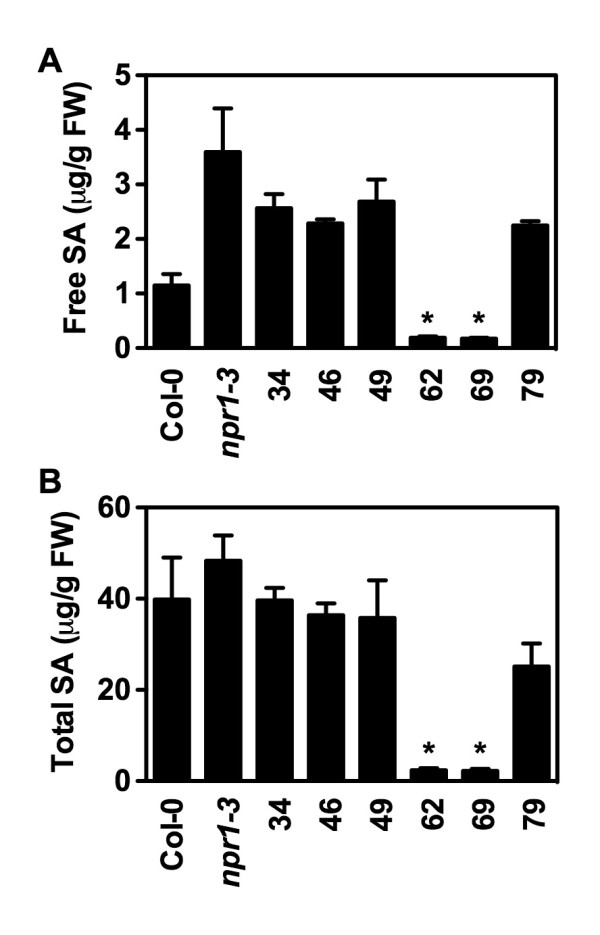
**SA levels accumulated in the *npr1 *suppressors determined by HPLC**. Free (**A**) and total (**B**) SA levels in *Psm *ES4326-infected *npr1 *suppressors were assayed using the HPLC-based method. Values are the mean of 4 samples with standard deviation. The experiment was repeated with similar results. The suppressors 62 and 69 accumulated significantly less SA than *npr1 *(*P < 0.0001).

**Figure 6:**
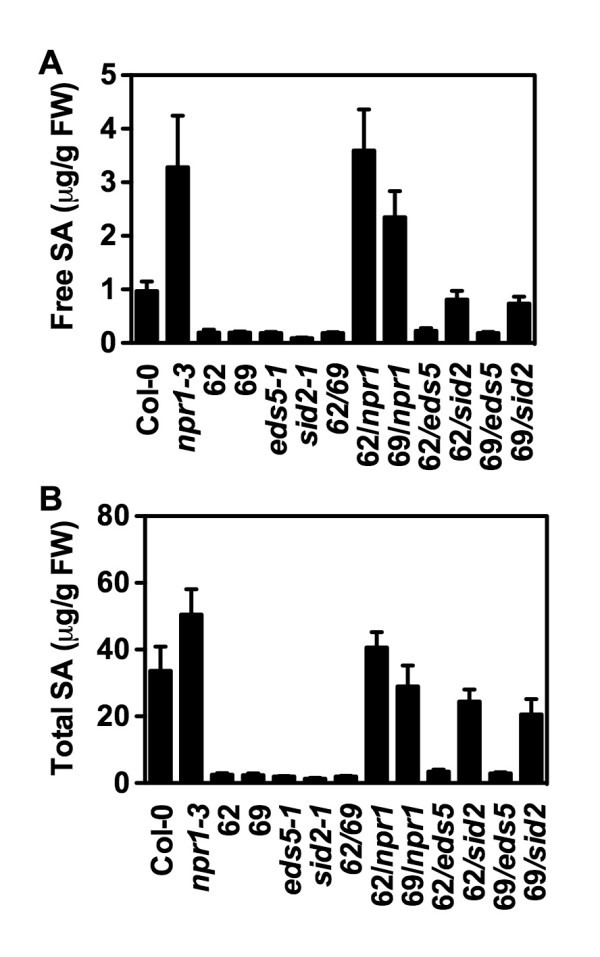
**SA levels accumulated in the F_1 _plants determined by HPLC**. Free (**A**) and total (**B**) SA levels in *Psm *ES4326-infected F_1 _plants of different crosses among 62, 69, *npr1*, *eds5*, and *sid2 *were assayed using the HPLC-based method. Values are the mean of 4 samples with standard deviation. The experiment was repeated with similar results.

To further confirm that the suppressors accumulate less SA than *npr1 *after pathogen infection, we measured SA levels in the suppressors using HPLC. As shown in Figure 5, after *Psm *ES4326 infection, all six suppressors accumulated lower levels of both free and total SA than *npr1*, suggesting that the suppressors contain mutations that modified the SA accumulation pathway in *npr1*. Interestingly, suppressors 62 and 69 accumulated very low levels of SA, similar to the previously characterized *eds5 *and *sid2 *mutant. To further characterize these two suppressors, we measured *Psm *ES4326-induced SA levels in the F_1 _plants of the following crosses: 62 × 69, 62 × *npr1*, 69 × *npr1*, 62 × *eds5*, 62 × *sid2*, 69 × *eds5*, and 69 × *sid2*. As shown in Figure 6, after *Psm *ES4326 infection, the F_1 _plants of 62 × 69 accumulated similar levels of SA as 62 and 69, whereas the F_1 _plants of 62 × *npr1 *and 69 × *npr1 *had similar amounts of SA as *npr1*, suggesting that 62 and 69 are allelic and contain a recessive mutation, since the 62 and 69 alleles will be heterozygous in the F_1 _Plants. Furthermore, the F_1 _plants of 62 × *eds5 *and 69 × *eds5 *accumulated similar levels of SA as *eds5*, whereas the F_1 _plants of 62 × *sid2 *and 69 × *sid2 *had similar amounts of SA as wild type, indicating that suppressors 62 and 69 are alleles of the *eds5 *mutant.

To identify the genetic mutations in 62 and 69, the open reading frames of the two alleles of *EDS5 *were sequenced. The allele 62 carries a transition mutation converting a TGG to a premature stop condon (TGA) at nucleotide 753 of the coding region, whereas the 69 mutation is caused by a G-to-A transition in the AG from the splice acceptor site in intron 5, which may lead to an abnormal splicing at the border of intron 5/exon 6. These results indicate that the mutant screen identified two new alleles of the previously isolated *eds5 *mutants [9].

### Pathogen Resistance Test for the SA Metabolic Mutants

**Figure 7:**
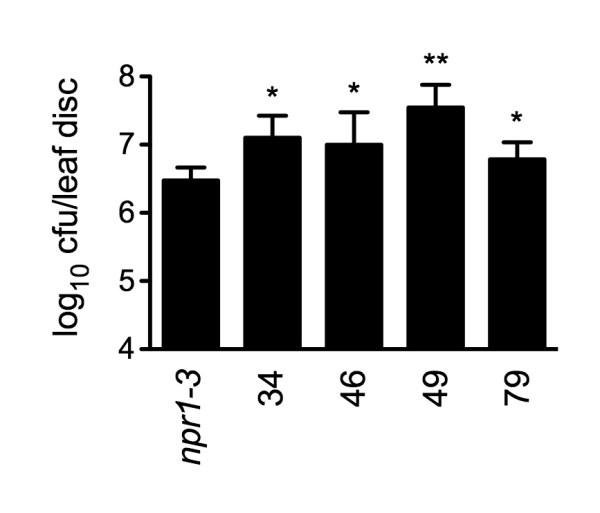
**Pathogen growth in the SA metabolic mutants**. Leaves of 4-week-old plants were inoculated with *Psm *ES4326 (OD_600 _= 0.0001). The in planta bacterial titers were determined 3 days postinoculation. Cfu, colony-forming units. Data represent the mean of 8 independent samples with standard deviation. *Psm *ES4326 grew significantly more in 34, 46, 49, and 79 than in *npr1-3 *(*P < 0.05, **P < 0.002). The experiment was repeated three times with similar results.

To test whether the genetic mutations in the SA metabolic mutants affect pathogen resistance, we inoculated *npr1-3 *and the four mutants, 34, 46, 49, and 79, with the bacterial pathogen *Psm *ES4326. The *eds5 *alleles, 62 and 69, were excluded since *eds5 *has been well characterized [9]. The in planta growth of *Psm *ES4326 was monitored three days after inoculation. As shown in Figure 7, *Psm *ES4326 grew significantly more in the SA metabolic mutants than in *npr1-3*. The growth of *Psm *ES4326 in 49 was 10-fold higher than in *npr1-3*. This result is significant since *npr1-3 *is already highly susceptible to *Psm *ES4326 [16], demonstrating that the high-throughput method developed in this study is valuable in identifying new components in the SA-mediated defense signaling pathway.

## Conclusions

Here we present a direct boiling protocol for the rapid estimation of SA from plant tissue using the SA biosensor *Acinetobacter *sp. ADPWH_*lux*. This protocol is much faster and less expensive than the previously described biosensor-based approaches [13,15]. The fast sample preparation procedure, which comprises inoculation of one leaf on each plant, collection of leaf discs, and boiling in LB, significantly reduced the time spent on inoculation, tissue collection, grinding and centrifugation. This method was not designed to accurately determine the SA concentration. Rather, it is intended to estimate SA levels for rapid genetic screens with significant reductions in cost and processing time. We acknowledge that SA levels induced by *Psm *ES4326 infection can vary quite a lot among individuals of the same genotype, depending on plant growth conditions. Additionally, this method may not be sensitive enough in some applications, such as time-course quantifications of SA levels with pathogen infection. However, the successful genetic screen for suppressors of the *npr1 *mutant has demonstrated the efficacy of this high-throughput strategy. We hope that the methodology presented in this study can help saturate the genetic screens for SA metabolic mutants, which in turn will facilitate a more thorough understanding of this important plant defense molecule [22,23].

## Methods

### Plant material and pathogen infection

The wild type used was the *Arabidopsis thaliana *(L.) Heynh. Columbia (Col-0) ecotype, and the mutant alleles used were *npr1-3 *[19], *eds5-1 *[9] and *sid2-1 *[8]. EMS mutagenesis was performed as described in [24]. Briefly, one gram of *npr1-3 *seeds were placed in 25 mL of 0.2% EMS (v/v) in a 50-mL Falcon tube and incubated on a rocking platform for 15 hours. After the seeds were washed eight times with water, they were suspended in 0.1% agarose and sown on soil. M_2 _seeds were collected in pools when the M_1 _plants reached to maturity. The M_2 _plants were germinated, transplanted to 96-pot trays, and then grown at 22~25°C under a 16 hr light/8 hr dark regime for three weeks. Infection of plants with *Psm *ES4326 was performed as described previously [25]. One leaf on each plant was infiltrated with a suspension of *Psm *ES4326 (OD_600 _= 0.001).

### Preparation of crude extract

Twenty-four hours after *Psm *ES4326 infection, a leaf disc was collected from each infected plant using a hole punch and placed in 200 μl of LB in a well of a 96-well PCR plate. The plate was then heated at 95°C for 20 min in a PCR machine and cooled down to room temperature.

### Detection of salicylic acid using *Acinetobacter *sp. ADPWH_*lux *and HPLC

An overnight culture of *Acinetobacter *sp. ADPWH_*lux *was diluted in 37°C LB (1:20) and grown for ~2 hrs at 200 rpm to an OD_600 _of 0.4. Using a multipipette, 50 μl of biosensor culture was added to each well in a 96-well black cell culture plate, and then 50 μl of the crude extract was added to each well and mixed by pipette action. The plate was incubated at 37°C for 1 hr without shaking before luminescence was read using a Veritas™ Microplate Luminometer (Promega Corporation, Sunnyvale, CA). Measurement of SA with HPLC was done as described by Verberne *et al*. [11]. Briefly, ~0.1 g tissues were ground in liquid nitrogen and extracted with 1 mL of 90% methanol. After centrifugation at 14,000 g for 10 min, the supernatant was transferred into a microcentrifuge tube. The pellet was extracted with 0.5 mL of 100% methanol and the supernatant was transferred to the same tube and dried in a speed vacuum to final volume of ~50 μL. The residue was resuspended to 500 μL with hydrolysis buffer (0.1 M sodium acetate buffer, pH 5.5). The mixture was equally split into two microcentrifuge tubes [one for free SA, the other for glucose conjugated SA (salicylic acid 2-*O*-β-D-glucoside or SAG)]. For SAG, 10 units of β-glucosidase were added to the tube. After incubation at 37°C for 1.5 hr, an equal volume of 10% TCA was added to both tubes. After centrifugation at 14,000 g for 10 min, the supernatant was transferred to a fresh tube and partitioned with 1 mL extraction solvent (1 ethylacetate: 1 cyclohexane). The top organic phase was transferred to a new tube, and dried in a speed vacuum to final volume ~25 μL. The residue was resuspended to 0.25 mL with 0.2 M sodium acetate buffer (pH 5.5). After centrifugation at 14,000 g for 10 min, the supernatant was used for HPLC analysis. The sample was eluted with 0.2 M sodium acetate buffer pH 5.5 in 10% methanol at a flow-rate of 0.80 mL/min.

### Statistical methods

All statistical analyses were performed with the data analysis tools (t-TEST: Two Samples Assuming Unequal Variances) in Microsoft Excel of Microsoft Office 2004 for Macintosh.

## Competing interests

The authors declare that they have no competing interests.

## Authors' contributions

GM, RC, YD, DS, and XZ performed the experiments. ZM designed the project, wrote the manuscript, and is the PI of the laboratory. All authors read and approved the final manuscript.

## Supplementary Material

Additional file 1 **Detailed protocol for identification of SA metabolic mutants using the direct boiling method**. Click here for file
