# Supplementary material for: A high-throughput method for isolation of salicylic acid metabolic mutants
Source: Plant Methods. 2010 Sep 23;6:21. doi: 10.1186/1746-4811-6-21 (PMC2949671; doi:10.1186/1746-4811-6-21)
Supplement: Additional file 1 — Detailed protocol for identification of SA metabolic mutants using the direct boiling method. [file 1746-4811-6-21-S1.DOC]

Detailed protocol for identification of SA metabolic mutants using the direct boiling method

1. Grow M_2_ plants in 96-pot trays until the plants are ready.
2. Inoculate one leaf on each plant with a suspension of the bacterial pathogen *Psm* ES4326 (OD_600_ = 0.001) by pressure infiltration using a 1-mL needleless syringe and wait for 24 hours.
3. Add 200 μl LB to each well in 96-well PCR plates using a multipipette. Harvest a leaf disc from each inoculated leaf using a hole punch and place into a corresponding well of the PCR plates.
4. Heat the samples at 95°C for 20 min in a PCR machine, and then cool down the extracts to room temperature.
5. Add 50 μl of a culture of the biosensor strain *Acinetobacter* sp. ADPWH_*lux* (OD_600_ = 0.4) to each well in black 96-well cell culture plates using a multipipette.
6. Add 50 μl of each leaf extract to a corresponding well and mix by pipette action. Incubate the plates at 37°C for 1 hr.
7. Pre-warm the microplate reader to 37°C and read luminescence.
